# Magnetorheological phase-change McKibben actuators for frequency-controlled actuation and high force output

**DOI:** 10.1038/s41598-026-57068-2

**Published:** 2026-06-19

**Authors:** Devansh Gupta, Sushma Santapuri

**Affiliations:** Department of Applied Mechanics, Indian Institute of Technology Delhi, Hauz Khas, New Delhi, Delhi 110016 India

**Keywords:** Soft robotics, Artificial muscle, McKibben actuators, Wireless actuation, System optimization, Engineering, Materials science, Physics

## Abstract

State-of-the-art soft robots face a critical trade-off: high-performance pneumatic actuators require bulky external compressors, whereas emerging smart-material actuators often lack sufficient force output. Here, we present a magnetorheological elastomer (MRE)-based phase-change McKibben actuator actuated via induction heating. The inclusion of ferromagnetic particles contributes to improved force output and thermal response of the elastomeric matrix. Furthermore, the design enables wireless operation, where the induction coil’s excitation frequency directly regulates the electromagnetic heating and actuation timing. A multiphysics modeling framework, integrating finite element electromagnetic analysis with analytical fluid-pressurization and structural models, guides the actuator design. Prototypes operating at 127 kHz and 150 kHz are fabricated and tested. They produce up to 70 N blocked force at a 23 g mass, representing an increase over prior phase-change actuators. The force output is 48% higher, with faster heating and cooling times compared to identically dimensioned hyperelastic actuators. Increasing the induction frequency to 150 kHz reduces the time to reach a 35 N load by nearly 25%. With heating times of 10–20 s and wireless operation, this actuator represents a promising platform for lightweight soft robotic systems.

## Introduction

Soft robotics is a branch of robotics that focuses on compliant and deformable components, aiming to replicate the flexibility, adaptability, and strength observed in biological organisms (e.g., octopuses, inchworm, human muscles)^1–4^. Unlike conventional robots, which are typically built using metals or hard plastics with limited degrees of freedom, soft robots are made of compliant materials with infinite degrees of freedom^5^. As a result, soft robots can be used to handle delicate tasks, adjust to unpredictable environments, and improve safety in human-robot interactions.

One of the main problems with designing soft robotic actuation systems is finding a way to achieve controlled motion while retaining material flexibility. Among the various approaches, pneumatic artificial muscles (PAMs) have attracted significant attention owing to their high power-to-weight ratios, large strain capabilities, and bio-mimetic contraction behavior^6–8^. The McKibben actuator is a widely recognised example. It has an inflatable inner bladder and a braided mesh sleeve around it. It produces axial contraction when it is pressurised internally^9–12^.

Despite their strong performance, traditional PAMs lack portability due to bulky components such as air compressors, pressurized tanks or control valves^13^. This limits their application in untethered soft robotic systems, where autonomy and mobility are essential. To overcome these challenges, phase-change actuators have been investigated, which utilize rapid liquid-to-gas transitions to generate pressure without bulky external sources^13–17^. These actuators often employ low boiling-point liquids (e.g., ethanol), which vaporize quickly under thermal stimulation to provide internal pressure and axial force suitable for untethered operation^13,16,18^.

Recent advancements in wireless heating techniques, such as induction heating, have enabled the untethered control of the actuation of phase-change materials. Induction heating uses alternating magnetic fields to create heat directly in conductive materials. This makes it possible to transfer energy wirelessly in an effective way^15,19,20^. These kinds of methods are very useful for soft robots that need to work without large power sources, bulky compressors, and pressure vessels^21–23^. Mirvakili et al.^13^ demonstrated the actuation of untethered pneumatic actuators through magnetically induced liquid-to-gas phase transitions. They reported a peak force output of 8 N and 20*%* strain. Ai et al.^24^ developed a miniaturized, untethered McKibben artificial muscle that is actuated using photo-thermally induced liquid to gas phase transitions, demonstrating remote high-energy-density soft actuation. The suggested actuator attains autonomy and adjustable performance by integrating the robustness and flexibility of pneumatic McKibben muscles with wireless phase-change actuation.

At the same time, incorporating smart materials into soft actuator designs has opened new possibilities for improving structural response and functionality^25^. Notably, magnetorheological elastomers (MRE) and dielectric elastomers (DE) are widely studied smart materials that exhibit field dependent behavior in various applications^26–30^.

In this work, we present the design and analysis of an MRE-based untethered McKibben actuator driven by phase change. A further contribution is the development of a numerical and analytical multiphysics framework to model the coupled processes of induction heating, heat transfer, fluid pressurization, and structural response to guide the design process. Through modeling-based optimization, the design parameters were refined and a proof-of-concept actuator was fabricated and experimentally analyzed. The MRE is used primarily as a passive composite wall that can influence thermal transport and large-strain structural response under phase-change actuation. Our actuator is capable of generating a maximum blocked force of 70 N (6.8 kg load) before failure at a mass of only 23 g. The overall weight of the setup is less than 600 g. Furthermore, by optimizing the fluid volume, ferromagnetic particle size, particle loading, and coil characteristics, the heating time for one cycle is reduced to 10–20 s. The actuator also avoids dependence on bulky pneumatic hardware or large power sources often required for conventional soft-actuation systems. Together with the multiphysics modeling framework, these results establish a pathway for lightweight, frequency-controlled, high-performance soft actuators suitable for wearable robotic augmentation.

## Methodology

**Fig. 1:**
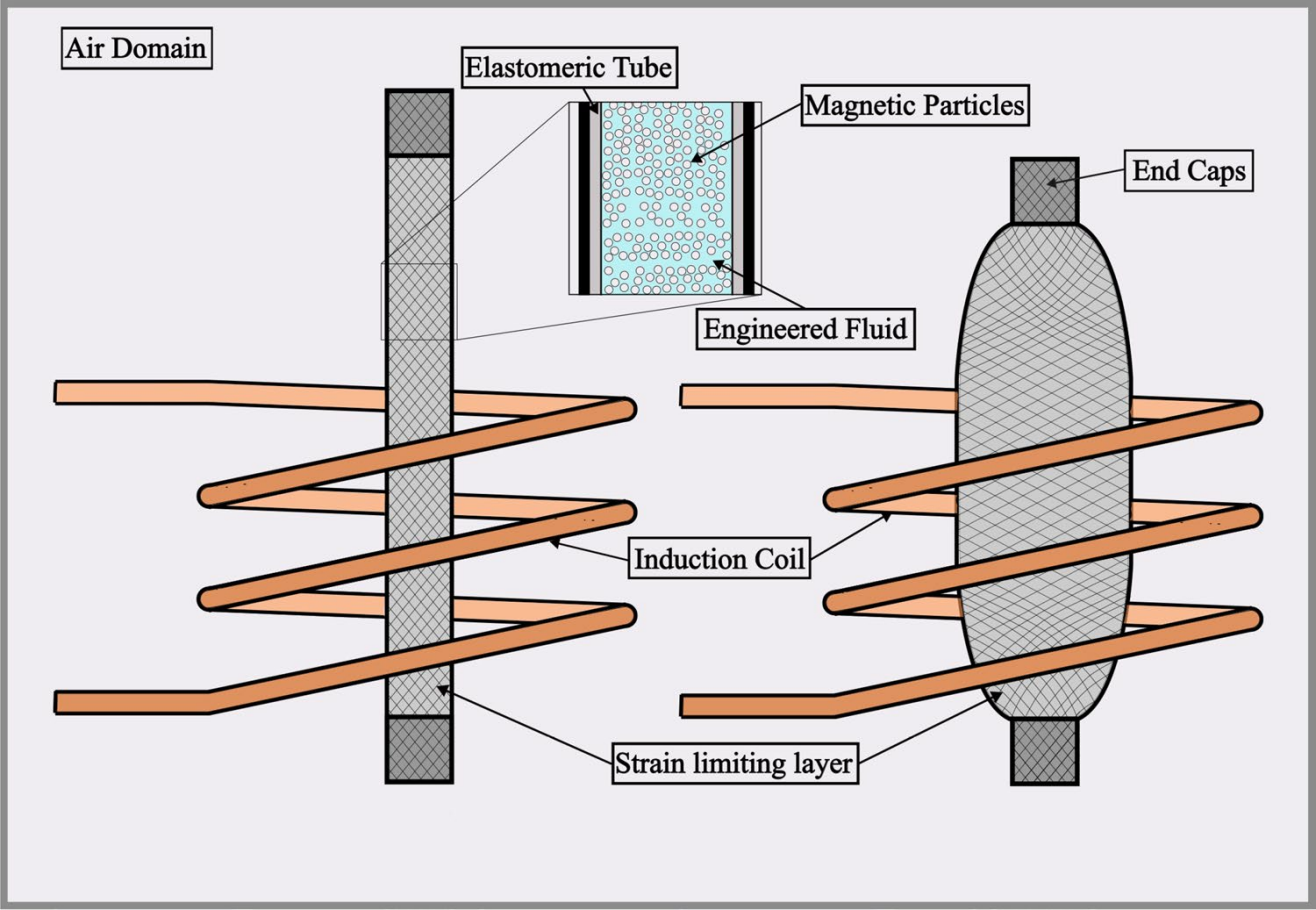
Schematic of the phase change artificial muscle actuator. The actuator is placed in the induction coil (left) and, upon activation, deforms due to fluid expansion (right).

The McKibben-type actuator considered in this work is schematically illustrated in Fig. 1. It consists of a tubular MRE enclosure, hermetically sealed at both ends, and containing a specific volume of low-boiling-point fluid with suspended ferromagnetic particles. This tube is housed within a braided sleeve, which constrains its radial expansion and converts it into axial contraction. When placed inside an induction coil, the actuator is subjected to a time-varying magnetic field, which induces eddy currents in the ferromagnetic inclusions via electromagnetic induction. The resultant Joule heating raises the local temperature within the fluid domain, eventually causing a phase transition from liquid to vapor. This phase change leads to a rapid volumetric expansion of the working fluid, generating internal pressure that inflates the tube. Due to the constraint imposed by the braided sleeve, the expansion is primarily converted into an axial contraction, thereby performing unidirectional actuation (An Indian patent application (no. 202611017534) has been filed for the magnetorheological elastomer based untethered artificial muscle actuator design). Modeling of the particle heating phenomenon is performed to guide the design. Subsequently, MRE and purely hyperelastic prototypes operating at different frequencies are fabricated and tested. In what follows, a multiphysics modeling framework is presented to study electromagnetic heating and its subsequent thermal transport phenomena.

### Governing equations

The electromagnetic field behavior in all the domains is described using the Maxwell’s equations:

1 $$\begin{aligned} \nabla \cdot {{\textbf {E}}}&= \frac{\rho _e}{\varepsilon _0}, \end{aligned}$$

2 $$\begin{aligned} \nabla \cdot {{\textbf {B}}}&= 0,\end{aligned}$$

3 $$\begin{aligned} \nabla \times {{\textbf {H}}}&= {\textbf {J}} + \frac{\partial {\textbf {D}}}{\partial t}, \end{aligned}$$

4 $$\begin{aligned} \nabla \times {{\textbf {E}}}&= -\frac{\partial {\textbf {B}}}{\partial t}. \end{aligned}$$

Here, ${\textbf {E}}$, ${\textbf {B}}$, and ${\textbf {H}}$ are the electric field intensity, magnetic flux density, and magnetic field intensity, respectively. $\rho _e$ is the free charge density, $\varepsilon _0$ is the permittivity of free space, ${\textbf {D}}$ is the electric field displacement, and ${\textbf {J}}$ denotes the current density. The constitutive equations relating ${\textbf {B}}$, ${\textbf {H}}$, ${\textbf {J}}$, and ${\textbf {E}}$ are given by

5 $$\begin{aligned} {{\textbf {B}}} = \mu {{\textbf {H}}}, \qquad {{\textbf {J}}} = \sigma {\textbf {E}}, \end{aligned}$$

where *μ* and *σ* are the magnetic permeability and the electrical conductivity of the medium, respectively.

To simplify the magnetic field analysis under the assumptions of linear, isotropic materials and negligible displacement currents, the magnetic vector potential ${\textbf {A}}$ is introduced such that

$$\begin{aligned} {\textbf {B}} = \nabla \times {\textbf {A}} . \end{aligned}$$

With this, the time-harmonic form of Maxwell’s equations leads to the magnetic vector potential equation

6 $$\begin{aligned} \nabla ^2 {\textbf {A}} + \omega ^2 \mu \epsilon {\textbf {A}} = j \mu \omega \sigma {\textbf {A}} - \mu {\textbf {J}}_{app}, \end{aligned}$$

where *ω* is the angular frequency of the excitation current, *ε* is the material permittivity, $j = \sqrt{-1}$, and ${\textbf {J}}_{app}$ is the externally applied current density in the induction coil.

Solving for ${\textbf {A}}$ allows computation of the induced eddy current density in the ferromagnetic domain^31^ as

7 $$\begin{aligned} \textbf{J}_{\text {eddy}} = -\sigma _{p} \frac{\partial \textbf{A}}{\partial t} = -j \sigma _{p} \omega \textbf{A}, \end{aligned}$$

where $\sigma _p$ is the conductivity of the suspended ferromagnetic particle. The resulting volumetric heat generation due to Joule heating is then obtained using

8 $$\begin{aligned} Q = \frac{|{\textbf {J}}|^2}{\sigma _{p}} . \end{aligned}$$

The output force of a pressure-driven McKibben artificial muscle can be expressed as a function of the differential pressure (*P*) between the ambient and the internal elastomeric tube, the initial braiding angle ($\theta _0$), the contraction strain (*\varepsilon*), and the initial radius of the actuator using the following relationship^32^

9 $$\begin{aligned} F(P,\varepsilon ) = (\pi r_0^2)P[a(1-\varepsilon )^2 - b], \end{aligned}$$

where $a = 3/tan^2(\theta _0)$ and $b = 1/sin^2(\theta _0)$. This formulation assumes full transmission of the internal pressure to the external braid and neglects effects arising from actuator stiffness and geometry changes at the end fittings. In the proposed actuator, internal pressure is generated by the liquid–vapor phase transition of the engineered fluid Novec 7100. The vapor pressure *P* of Novec 7100 as a function of absolute temperature $T_n$ (in Kelvin) is given by the Clausius-Clapeyron equation^33^

10 $$\begin{aligned} ln~P = 22.415 -\frac{3641.9}{T_n}. \end{aligned}$$

### Numerical simulation setup

The numerical simulation workflow is divided into two key components: (1) coil characterization, aimed at evaluating the optimal geometric parameters of the induction coil, and (2) particle-level heat loss analysis, focused on understanding the heat generation and dissipation behavior of ferromagnetic particles under alternating electromagnetic excitation. The numerical framework is intended as a component-level design tool rather than a fully coupled predictive multiphysics model. Owing to the strong coupling between electromagnetic excitation, transient heat transfer, phase-change dynamics, and fluid flow within the PCM domain, a fully resolved model would be computationally expensive and beyond the scope of the present study. Accordingly, the simulations are employed primarily to optimize key design parameters (e.g., particle size and excitation frequency) and guide subsequent experimental validation.

#### Coil characterization

The induction coil used in this study operates by converting a 24 V DC input into a high-frequency alternating current through the Zero Voltage Switching (ZVS) mechanism. This alternating current, when passed through the copper coil, generates an alternating magnetic field. Any ferromagnetic material placed within this field undergoes induced eddy currents, resulting in Joule heating through $I^2R$ losses within the material domain.

**Fig. 2:**
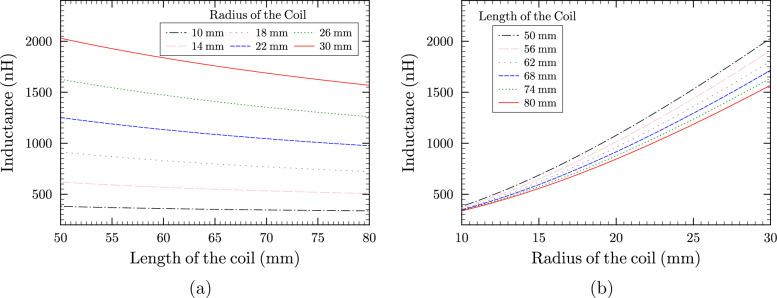
Coil analysis using simulations: Variation of coil inductance with (a) coil length, and (b) coil radius.

A high-power induction coil circuit, rated for up to 900 W, is used to ensure rapid heating and actuation. The performance and efficiency of the induction coil are highly dependent on the physical parameters of the coil like its radius, length, number of turns, and tube gauge, which influence the coil’s overall inductance and, by extension, its resonant frequency. To predict the optimal coil dimensions for achieving target inductance values and corresponding resonant frequencies, the induction coil was modeled in COMSOL Multiphysics. A parametric study was conducted on a helical copper tube geometry by varying its radius from 10 mm to 30 mm and length from 50 mm to 80 mm. The number of turns was fixed at 6.5 for all simulations. The computed inductance values across the parameter space is plotted in Fig. 2a and Fig 2b, which provides insight into how geometric changes affect the coil’s inductance.

**Fig. 3:**
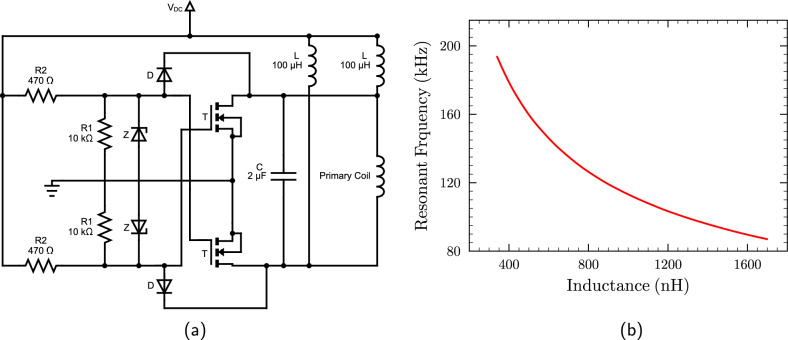
(**a**) Circuit diagram for the high-power ZVS induction heating system. Component specifications are detailed in Table 1. (**b**) LTspice simulation showing the variation of resonant frequency with coil inductance. Lower inductance results in higher frequencies.

To further understand the electrical response of the designed coil, circuit-level simulations were performed in LTspice. The circuit schematic is shown in Fig. 3a, and the specific components used are listed in Table 1. The simulation results, presented in Fig. 3b, show that a decrease in inductance leads to an increase in resonant frequency.

**Table 1:** Component Specifications for the ZVS Induction Heating Circuit.

Symbol	Description
C	6 *×* 330nF, 600 V, AC Capacitors
L	100 *μ*H, 15 A, Ferrite Core Inductors
D	UF4007 ultrafast diode
T	IRFP250N MOSFET
Z	12 V, 1 W Zener Diode
R1	10 k*Ω* Resistor
R2	470*Ω*, 5 Watt Resistor
$V_{DC}$	24 V DC Input

To achieve a coil geometry with practical dimensions that are feasible to fabricate and integrate withing the experimental setup, the inductance of the coil was constrained to the range of 500–900 nH. This range ensures a resonant frequency in the desired band (120–150 kHz) while allowing sufficient design flexibility in terms of the number of turns, coil diameter, and spacing.

#### Coil and particle simulation

This section focuses on analyzing the heating characteristics of ferromagnetic particles placed inside an induction coil, with the goal of understanding the influence of particle size, coil frequency, and particle volume fraction on volumetric heat generation, through simulations. The governing equations (6)–(8) are solved using the finite element software COMSOL Multiphysics. The simulation domain consists of a helical induction coil modeled as a hollow copper tube and a 3D array of spherical iron particles. The dimensions of the coil used in the simulations are detailed in Table 2. The 127 kHz frequency coil was modeled corresponding to an inductance of approximately 800 nH, as determined from coil characterization (see Section “Coil characterization”). The material properties of the components are mentioned in Table 3.

**Table 2:** Dimensions of the Induction Coil.

Parameter	127 kHz	150 kHz
Number of turns	6.5	5.5
Radius	18.5 mm	15.75 mm
Tube outer radius	2.38 mm	2.38 mm
Tube inner radius	1.78 mm	1.78 mm
Pitch	10.61 mm	11.82 mm

**Table 3:** Material Properties of components of the actuator.

Domain	Parameter	Value	Unit
Fluid	Heat capacity	1183	*J/(Kg· K)*
	Boiling point	61	$\vphantom{0}^\circ C$
	Density	1510	$kg/m^3$
Particle	Relative permeability	9	
	Relative permittivity	1	
	Electrical conductivity	$1.12 \times 10^7$	*S*/*m*
	Density	7870	$kg/m^3$
	Weight	3	*g*
Copper Coil	Relative permeability	1	
	Relative permittivity	1	
	Electrical conductivity	$5.998 \times 10^7$	*S*/*m*
Air	Ambient temperature	30	$\vphantom{0}^\circ C$

The coil-particle system was embedded in a cuboidal air domain, which was enclosed with an infinite element layer at its boundaries to simulate unbounded space and minimize reflection. The electromagnetic simulation utilized the Magnetic and Electric Fields physics, with perfect magnetic conductor (PMC) boundary conditions imposed on the outer surfaces of the air domain to approximate magnetic insulation. A sinusoidal current (obtained from circuit analysis), was applied to the coil as the excitation source.

For meshing, a user-controlled mesh strategy was adopted to ensure high accuracy in regions of interest while maintaining computational efficiency. The copper coil was discretized using a swept mesh with an extremely fine element size to resolve current distribution. The ferromagnetic particles were meshed using finer sized tetrahedral elements to capture localized heating gradients. The surrounding air domain was assigned a coarser tetrahedral mesh, and the infinite element layer was meshed using a swept mesh with extremely coarse discretization. The simulations were conducted in the frequency domain, varying several key parameters to evaluate their influence on the volumetric power loss density (W/m$\vphantom{0}^3$) within the ferromagnetic particles.

### Fabrication and experimental analysis of the phase change actuator

**Fig. 4:**
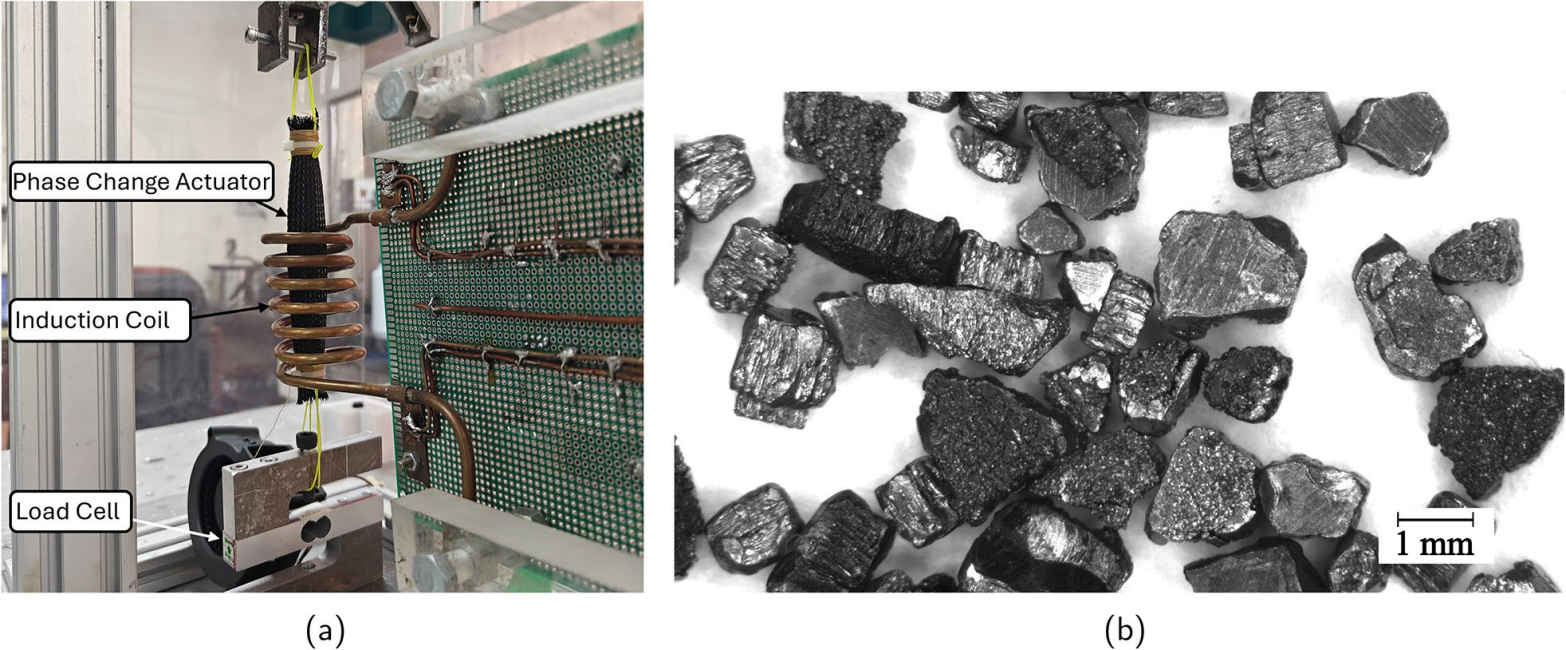
(**a**) Actuator test setup: The phase change actuator is placed inside the induction coil, suspended from the top and attached to the load cell at the bottom. (**b**) Microscope image of the dispersed ferromagnetic particles (Iron, Sigma Aldrich).

The actuator is made using a Magnetorheological Elastomer (MRE) tube filled with a low boiling point engineered fluid (Novec 7100 Engineered Fluid) with suspended ferromagnetic particles. The MRE tube is sealed at both ends with 3D printed end caps. The sealed tube is then placed inside a braided sleeve, made of inextensible nylon strings. The ends of the tube are sealed with the braided sleeve using threads, glue, and fasteners. The prepared actuator is then placed inside an induction coil and the ends are attached to a fixed end and a load cell (Fig. 4a). Each component of the setup is discussed in detail in the subsequent subsections.

#### Actuator fabrication

The actuator fabrication is done in three steps: fabrication of MRE tube, 3D printing of end caps, and integration with braided sleeves and fasteners. The resulting actuator is tested using the experimental setup described below.

To fabricate the MRE tube a removable mold is prepared to form the annular geometry. Two flat acrylic plates are joined using nuts and bolts, followed by drilling a 12 mm through-hole at their intersection. A solid cylindrical rod serves as the core. In this case, an iron rod with a diameter of 10 mm is used. The mold is then sealed with 3D-printed end caps, which hold the core material in place at the center. A liquid silicone elastomer (SMOOTH-ON Dragon Skin 10) is selected as the matrix material for the actuator. The two-part silicone is mixed with Fe$\vphantom{0}_3$O$\vphantom{0}_4$ nanoparticles (Iron(II,III) oxide, Sigma Aldrich) and poured into the mold. The filled mold is then placed in a vacuum degassing chamber at a vacuum pressure of 730 mmHg for 5 minutes to remove entrapped air. The mold is subsequently removed from the chamber, and the core rod is inserted and centered using 3D-printed end caps. The mixture is allowed to cure at room temperature for 6 hours. Upon completion of curing, the two-part acrylic mold is disassembled, and the core rod is extracted, resulting in a fabricated MRE tube.

The MRE tube is first sealed at one end using fasteners and a 3D printed stopper. A low-boiling point engineered fluid (Novec 7100) is then poured into the tube, and Fe granules (Iron, Sigma Aldrich) of 10–40 mesh size (corresponding to a particle diameter range of approximately 2.00 mm to 0.40 mm) are suspended inside the engineered fluid. This size distribution, which yields an effective mean particle diameter of *1285~μ m* with a standard deviation of *279.3~μ m*, was verified against the manufacturer’s data sheet^34^ and confirmed via optical microscopy (Fig. 4b). The tube is sealed at the other end, and is subsequently enclosed within a braided sleeve and secured with fasteners.

#### Induction coil

The circuit layout shown in Fig. 3a is fabricated using commercially available components. Care is taken during soldering to ensure electrical integrity and mechanical stability. The primary coil, essential to system operation, is fabricated from a copper tube wound around a custom 3D-printed mold with predefined helical grooves (to ensure uniform spacing and tight winding along the coil length). The overall dimensions of the coil are listed in Table 2. Following fabrication, the coil is connected to a DC power supply, and a constant voltage of 24 V is applied. This value is determined from simulations and preliminary tests to balance performance and operational safety. The induction coil, along with all its components, weighs only about 500 g. A cooling fan is attached to the induction coil, which cools the actuator down when the induction coil is turned off. The cooling fan system weighs around 80 g.

#### Test setup

The induction coil is operated via DC relays controlled through a custom LABVIEW interface enabling real-time monitoring and automation. A calibrated 100 N load cell, mounted at one end of the actuator, measures the axial force. The analog output is continuously acquired and processed in LABVIEW. The system is programmed to maintain cyclic loading within a defined force window by deactivating the coil and activating a cooling fan when the load reaches 35 N, and reactivating the coil once the force falls below 10 N. This closed-loop control ensures stable, repeatable operation and facilitates characterization of the actuator’s thermo-mechanical response and fatigue behavior.

### Analytical heat transfer and thermodynamic modeling

To estimate the thermodynamic response of the actuator, an analytical lumped-capacitance heat transfer model was developed. The internal Novec 7100 working fluid is treated as a uniform thermal mass that receives thermal energy from the electromagnetically heated particles while simultaneously losing heat to the ambient environment through the cylindrical actuator wall. The model combines geometry-based thermal resistance terms with calibrated loss parameters obtained from the cooling response, to estimate the dominant thermal trends. The general transient energy balance for the internal fluid is given by

11 $$\begin{aligned} m_l C_{pl} \frac{dT}{dt} = P_t - q_{loss}, \end{aligned}$$

where $m_l$ is the mass of the fluid, $C_{pl}$ is the specific heat capacity, *T* is the fluid temperature, $q_{loss}$ is the rate of heat dissipation to the environment, and $P_t$ is the total heat generated in the ferromagnetic particles, calculated as

$$\begin{aligned} P_t = \frac{1}{\rho _p} m_p Q_{vl}. \end{aligned}$$

Here, $Q_{vl}$ is the volumetric loss density in the ferromagnetic particles, $m_p$ and $\rho _p$ are the particle mass and density, respectively. The environmental heat loss is governed by a series thermal-resistance network consisting of the conductive resistance through the actuator wall ($R_{cond}$) and the convective resistance at the outer surface ($R_{conv}$). For a cylindrical tube of inner radius $r_i$, outer radius $r_o$, and active length *L*, these resistances are defined as

12 $$\begin{aligned} R_{cond} = \frac{\ln (r_o/r_i)}{2\pi k L}, \quad R_{conv} = \frac{1}{2\pi r_o L h}, \end{aligned}$$

where *k* is the bulk thermal conductivity of the wall material and *h* is the convective heat transfer coefficient. The total heat loss rate is therefore expressed as

13 $$\begin{aligned} q_{loss} = \frac{T - T_0}{R_{total}} = \frac{T - T_0}{R_{cond} + R_{conv}}, \end{aligned}$$

where $T_0$ is the ambient temperature. The inclusion of iron particles in the MRE tube increases the effective thermal conductivity (*k*) relative to pure hyperelastic silicone, thereby lowering $R_{cond}$ and altering the thermal dynamics of the actuator.

#### Heating phase dynamics

During the active actuation phase, the alternating magnetic field is applied and Joule heating is generated continuously within the particles ($P_t> 0$). In this state, the outer surface of the actuator is exposed to ambient air, and heat dissipation is driven strictly by natural convection. Consequently, the time taken by the fluid to reach a prescribed temperature $T_f$ can be calculated by solving the governing Equation (11), and is found to be

14 $$\begin{aligned} t = m_l C_{pl}R_{total} \ln \bigg (\frac{P_t R_{total}}{P_t R_{total}+T_0 - T_f}\bigg ). \end{aligned}$$

The fluid temperature rises until the saturated vapor pressure reaches the threshold required for mechanical actuation which can be calculated using Eq. (10).

#### Cooling phase dynamics

Following actuation, the electromagnetic field is deactivated ($P_t = 0$), and an external cooling fan is engaged to return the actuator to its resting state. This active cooling shifts the boundary condition to forced convection, drastically increasing the external heat transfer coefficient and minimizing $R_{conv}$. The time taken by the actuator to reach a temperature $T_s$, starting from an initial fluid temperature of $T_f$, can be found by solving the governing Equation (11), and is found to be

15 $$\begin{aligned} t = m C_p R_{total} \ln \bigg (\frac{T_f - T_0}{T_s - T_0}\bigg ). \end{aligned}$$

For a pure silicone tube with very low thermal conductivity (*k ≈ 0.22* W/m*·*K), $R_{cond}$ acts as a dominant thermal bottleneck. Even if the cooling fan reduces $R_{conv}$, the overall cooling rate remains constrained by the insulating wall. In contrast, the enhanced thermal conductivity of the MRE tube minimizes $R_{cond}$, allowing the internal fluid to fully capitalize on the forced external convection, thereby facilitating a faster cooling response.

## Results and discussion

### Numerical simulation results

The electromagnetic heating characteristics of the ferromagnetic particles are performed to analyse the effects of particle size, coil frequency, and particle volume fraction on the volumetric heat generation rate.

#### Frequency dependence of particle heating

**Fig. 5:**
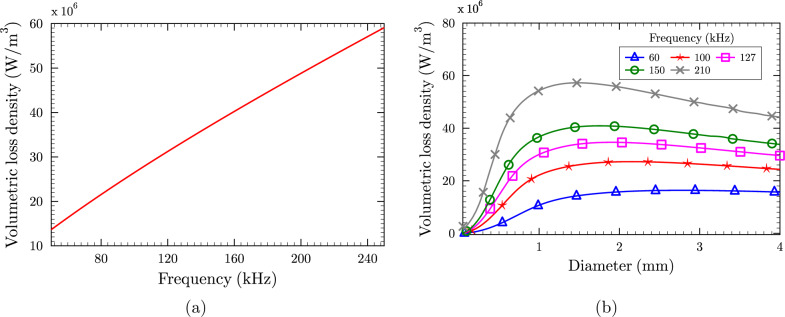
(**a**) Variation in volumetric power loss density with coil frequency for a 1285 *μ*m diameter iron particle placed at the center of the induction coil. (**b**) Volumetric power-loss density as a function of particle diameter at various excitation frequencies. The loss density increases with diameter at small sizes, reaches a maximum at an intermediate diameter (*≈*1–2 mm), and then gradually decreases. Higher frequencies yield larger peak losses and a stronger size dependence.

To examine the effect of induction frequency on particle heating, a single iron particle with a diameter of 1285 *μ*m was placed at the center of the coil. A parametric frequency sweep from 50 kHz to 250 kHz was performed while keeping the coil current as obtained from LTSpice simulations (102 A). The resulting volumetric loss density in the particle was evaluated and plotted as a function of frequency (Fig. 5a). As expected from eddy current theory, the loss density increases monotonically with frequency, confirming that higher frequencies result in more efficient electromagnetic heating for a given particle size. These numerical predictions are consistent with our experimental results (Section “Experimental results”), which demonstrated that increasing the frequency from 127 kHz to 150 kHz reduced the actuation time by nearly 25%.

#### Influence of particle size on heat generation

A separate study was conducted to investigate how particle size influences volumetric heat generation. The particle diameter was varied from 100 *μ*m to 4 mm while simulations were repeated for multiple coil frequencies. As shown in Fig. 5b, the volumetric loss density increases with diameter up to an optimum diameter (*≈*1–2 mm) and then decreases for larger particles. The initial rate of increase and the peak loss are substantially larger at higher frequencies, indicating a strong frequency–size coupling effect in electromagnetic heating performance. The decline beyond the peak is consistent with reduced volumetric heating for oversized particles due to the skin effect. For the operating frequency range of 120–150 kHz, a particle diameter of approximately 1.5mm yields the maximum volumetric loss density.

#### Effect of particle volume fraction on heating efficiency

To study the collective impact of particle concentration, a series of simulations were conducted by placing a 3D array of spherical iron particles of diameter 1285 *μ*m inside the coil. The number of particles was varied by constructing cubic arrays of size $n \times n \times n$, where *n* ranged from 1 to 8. This resulted in a progressive increase in total particle volume from $1.11 \times 10^{-9}\text {m}^3$ to $5.68 \times 10^{-7}\text {m}^3$. The average volumetric loss density was then computed for the particle array as a function of array size. It is observed that the volumetric loss density exhibits only a weak dependence on particle volume fraction. Therefore, modeling a single particle as a representative unit is assumed to provide a reasonable estimate of volumetric loss density of a multi-particle system.

It is important to define the limits of the magnetorheological effect within this specific operating regime. The electromagnetic simulations indicate that the peak alternating magnetic field intensity generated by the induction coil is approximately 10 kA/m. Within the present operating regime, no measurable field-induced change in the actuator’s base shear modulus was observed, so any such variations are considered negligible^35^. Consequently, within the scope of this study, the embedded ferromagnetic nanoparticles are contributing to thermal transport and to the composite wall response under transient pressurization, while the liquid-to-gas phase change remains the primary driver of actuation.

### Experimental results

The experimental procedures and data acquisition strategies follow the methodology outlined in Section "Fabrication and experimental analysis of the phase change actuator", with parametric variations implemented to assess the influence of material composition, fluid volume and excitation frequency. A control actuator is fabricated using a hyperelastic tube in place of the MRE tube for direct comparison under identical conditions. Cyclic loading–unloading tests are performed on the hyperelastic actuator containing 3 g of suspended ferromagnetic particles inside an induction coil. Particles with an average diameter of 1285 *μ*m are selected, as they exhibit good volumetric loss density within the frequency range of 127–150 kHz, as predicted by the simulations in Section "Influence of particle size on heat generation".

**Fig. 6:**
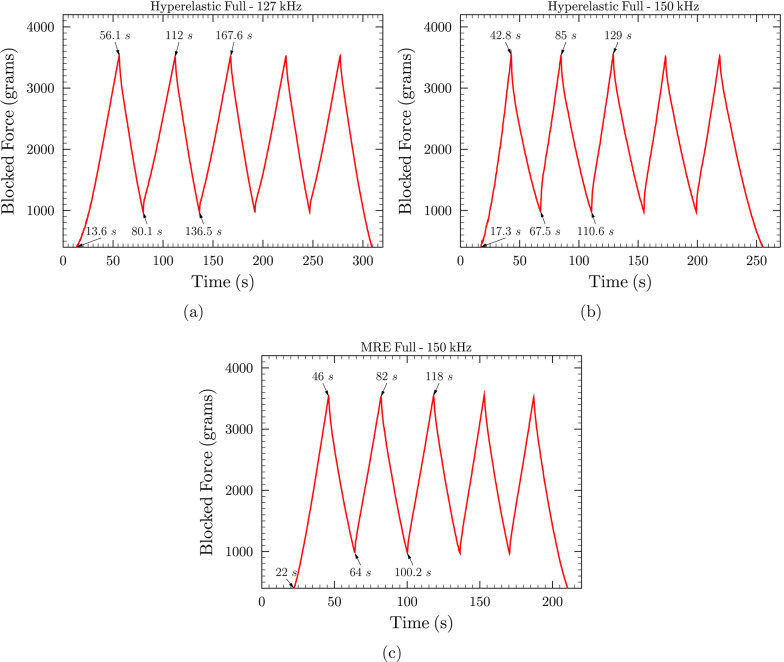
Cyclic loading-unloading plots for (**a**) hyperelastic actuator under 127 kHz frequency coil, (**b**) hyperelastic actuator under 150 kHz frequency coil, and (**c**) MRE actuator under 150 kHz frequency coil. The timestamps annotated at the peaks and troughs represent absolute elapsed times.

*Effect of Induction Coil Geometry*: As described in Section “Coil characterization”, the resonant frequency of the induction system is tuned by modifying the primary coil geometry. A coil that achieves a resonant frequency of 127 kHz is fabricated, along with a coil with altered dimensions that achieves 150 kHz resonance. The dimensional parameters of the coils are listed in Table 2. These coils are used to actuate the hyperelastic actuator completely filled with the engineering fluid and 3 g of suspended particles. The corresponding force responses are shown in Figs. 6a and 6b. Among these configurations, the hyperelastic actuator with the coil operating at 150 kHz exhibits a faster response, completing five loading–unloading cycles in a shorter duration. This result is consistent with the simulation results in Section "Frequency dependence of particle heating", which indicate a higher volumetric loss density at higher excitation frequencies.

*Effect of MR Elastomer*: To isolate the contribution of the MRE material, a hyperelastic baseline actuator and an MRE actuator were fabricated and tested under identical controlled parameters: initial braiding angle, fluid volume, suspended particle loading (3 g of 1285±279.3 *μ*m Fe particles), and induction coil frequency (150 kHz). The sole variable was the elastomeric tube material. The cyclic loading-unloading tests are performed and are plotted in Fig. 6. The thermal response during heating and cooling phases for these actuator configurations, specifically in the first and third actuation cycles, is presented in Fig. 8. In the first heating cycle the hyperelastic actuator reaches the target blocked force marginally faster than the MRE actuator; however, by the third cycle both materials require similar times to attain 3500 g load. Cooling behaviour differs: during the first cycle the MRE actuator cools more rapidly. This enhanced thermal diffusivity is a well established property of particle-filled composite materials. Pure silicone rubber acts as a thermal insulator with a baseline thermal conductivity of approximately *0.22 - 0.26~W/(mK)*^36^. By contrast, the embedded iron particles are highly conductive. Prior studies on magnetorheological elastomers have demonstrated that the dispersion of iron-based microparticles within a silicone matrix significantly increases the bulk thermal conductivity of the composite^37^. The particle-filled wall therefore provides greater thermal bridging, allowing the MRE actuator to transfer heat to the ambient environment more effectively than the pure hyperelastic tube. A similar trend is observed in the third cycle, with comparable cooling times for the full-volume hyperelastic and MRE actuators.

**Fig. 7:**
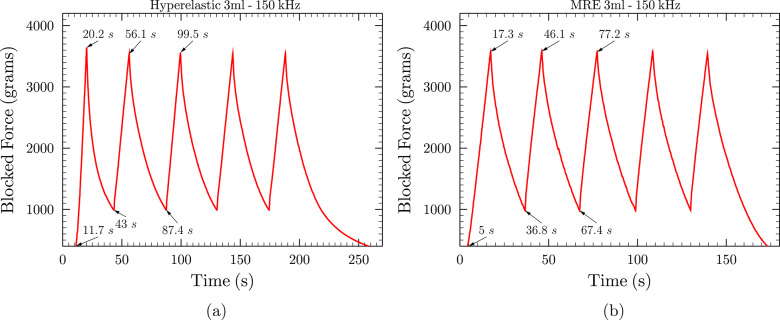
Cyclic loading-unloading plots for (**a) **hyperelastic actuator with 3 ml fluid under 150 kHz frequency coil, and (**b**) MRE actuator with 3 ml fluid under 150 kHz frequency coil. The timestamps annotated at the peaks and troughs represent absolute elapsed times.

**Fig. 8:**
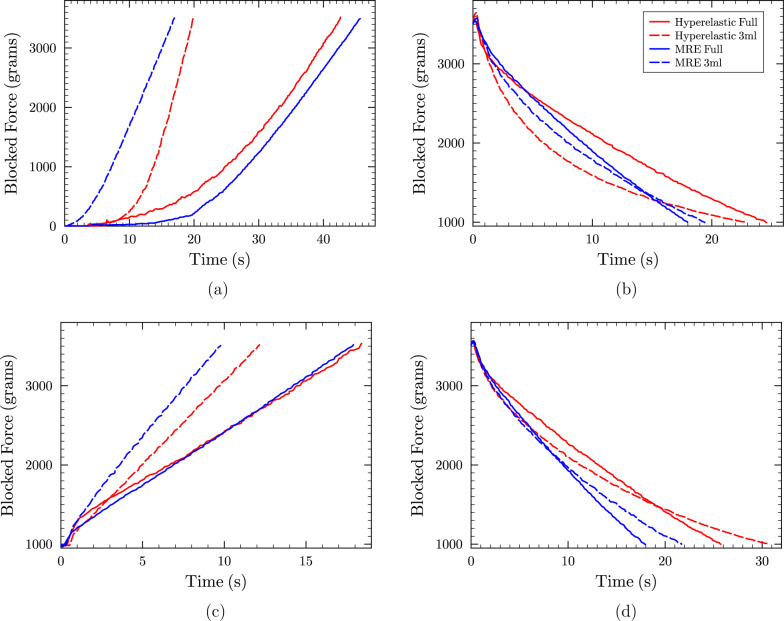
Time vs load plots for all the actuator configurations under a 150 kHz frequency induction coil for (**a**) heating during the first cycle, (**b**) cooling during the first cycle, (**c**) heating during the third cycle, and (**d**) cooling during the third cycle.

*Effect of Working Fluid Volume*: Subsequently, we investigate a design variation in which the actuator is partially filled with 3 ml of engineered fluid instead of being completely filled. The corresponding cyclic response is shown in Fig. 7, with heating and cooling phases specifically during the first and third actuation cycles presented in Fig. 8. Reducing the fluid volume is found to accelerate both heating and cooling, as less thermal energy is required to reach the boiling point, and the increased vapor volume condenses more rapidly during the cooling cycle. Consequently, the 3 ml configuration exhibits shorter rise times and reduced thermal time constants, demonstrating that actuator response time can be tuned through the initial fluid volume.

### Pneumatic characterization

**Fig. 9:**
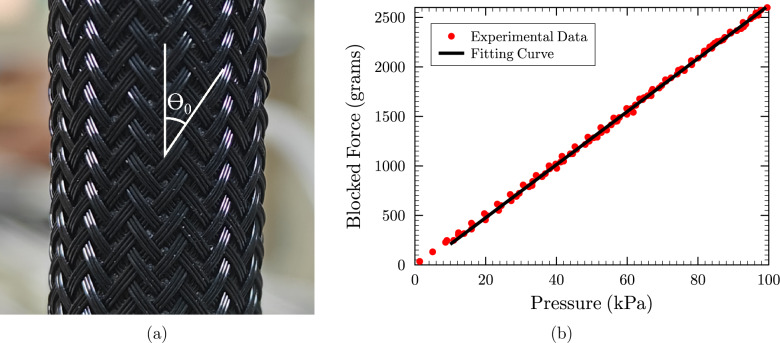
(**a**) Close-up image of the braided nylon sleeve used to determine the initial braiding angle. (**b**) Blocked force output of the MRE McKibben actuator as a function of applied internal pneumatic pressure. The data isolates the structural performance from the thermal phase-change generation.

To explicitly isolate the structural mechanics of the actuator from the electromagnetic phase-change dynamics, a decoupled pneumatic characterization is performed. First, the initial braiding angle ($\theta _0$) of the fabricated MRE McKibben actuator is measured and found to be 38$\vphantom{0}^\circ$, as shown in Fig. 9a. The actuator is then connected to an external, regulated pneumatic pressure source to bypass the internal induction heating mechanism.

The actuator is subjected to controlled internal pressurization while the blocked force is continuously recorded. The resulting pressure versus blocked force relationship is presented in Fig. 9b. This decoupled characterization provides a direct mapping between the internal fluid pressure and the mechanical output of the MRE McKibben actuator.

From this experimental profile, we identify that generating a target load capacity of 3500 g requires an internal fluid pressure of 132 kPa. Establishing this pressure requirement allows us to link the mechanical performance back to the internal thermodynamics of the Novec 7100 working fluid. Using the saturated vapor pressure relationship defined in Equation (10), the temperature required to achieve this specific internal vapor pressure is calculated to be $89^\circ$C. Similarly, a blocked force of 1000 g is achieved at an internal vapour pressure of 39.5 kPa, and the temperature is found to be $71.8^\circ$C.

### Analytical heat transfer calculations

Using Table 3, the total time required for the fluid to reach $89^\circ \text {C}$ from $30^\circ \text {C}$ is now estimated using Equation (14). Simulations assuming isolated particles with a diameter of 1285 ± 280 *μ*m and incorporating a natural convective heat transfer coefficient of $15~{W}/{m}^2{K}$ predict a heating time of approximately 47 ± 4 s. This value is broadly consistent with the experimental observations, where the actuator fully filled with the engineered fluid produced an output blocked force of 3500 g in 46 s. Possible sources of the remaining minor discrepancy include the neglect of fluid heat transfer effects and the omission of particle agglomeration in the heat-loss analysis, both of which are difficult to capture in the isolated-particle FEA model.

To quantify the cooling advantage of the MRE actuator, the cooling time was calculated using the forced-convection framework discussed in Equation 15. After deactivating the induction coil and switching on the external cooling fan, the forced convective heat transfer coefficient at the actuator surface increases to approximately 70 $W/m^2 K$. For the pure hyperelastic baseline actuator (*k = 0.18~W/mK*), the high conductive thermal resistance ($R_{cond}$) of the silicone wall creates a bottleneck, resulting in a calculated cooling time of 24.5 s to return to the baseline pressure of 39.5 kPa. In contrast, the MRE actuator benefits from the higher thermal conductivity of the ferrous particle-filled wall ($k \approx 0.9\,W/mK$)^38^. By reducing the internal thermal resistance, the presence of MRE allows the internal fluid to better utilize the forced external convection, reducing the required cooling time to 18.6 s. This reduction in convective recovery time supports the MRE’s functionality, and indicates that the ferromagnetic inclusions can increase the allowable cyclic operating frequency of the actuator.

### Maximum load capacity

**Fig. 10:**
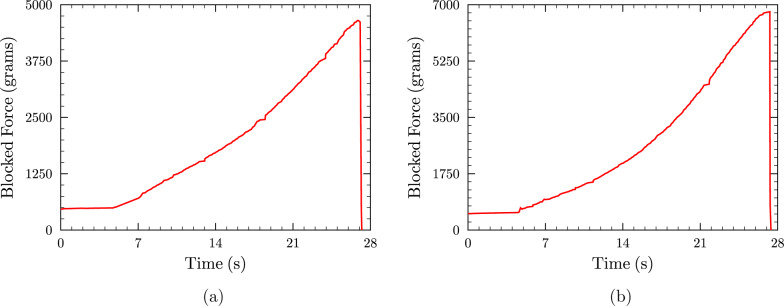
Maximum axial load for (**a**) the hyperelastic actuator, and (**b**) the MRE actuator.

**Fig. 11:**
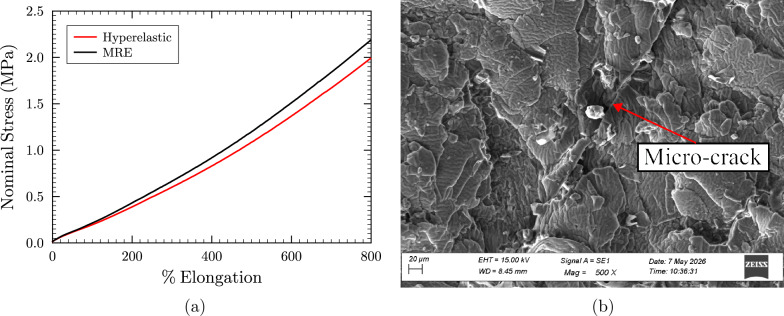
(**a**) Uniaxial tensile stress-elongation curves for the pure hyperelastic silicone and the composite MRE, measured according to the ASTM D412 standard. The curves illustrate a clear trend where the MRE exhibits a higher effective modulus due to the rigid iron inclusions. (**b**) SEM image of the MRE tube showing the presence of a microcrack.

We now evaluate the maximum axial load capacity of the hyperelastic and MRE actuators by subjecting them to maximum load test using the 150 kHz coil with 3 g of suspended ferromagnetic particles. As shown in Fig. 10, the hyperelastic actuator reaches 4650 g, while the MRE actuator with identical dimensions achieves 6780 g. We performed standard ASTM D412 tensile tests on both the pure silicone and the MRE composite. The resulting stress-strain curves (Fig. 11a) indicate a slightly higher effective modulus for the MRE sample than for the baseline silicone. The higher blocked force of the MRE actuator is not attributed solely to increased stiffness. Rather, the MRE wall appears to better maintain structural integrity at large deformation and resist localized bulging under pressurization.

In a traditional McKibben-type actuator, a stiffer inner tube would generally resist radial expansion and reduce contraction stroke, although its effect on blocked force depends on the balance between pressure containment and deformation constraints. In the present phase-change system, however, the composite wall may provide an advantage under high internal pressure by better controlling the large-strain deformation mode and limiting localized ballooning. This behavior is consistent with a more efficient translation of internal expansion into axial contraction. The performance of the MRE actuator is compared with already existing McKibben actuators of a similar kind in Table 4.

*Failure Mechanisms*: Both the hyperelastic and MRE actuators failed primarily by tube rupture, indicating that the chamber wall is the main load-bearing element. A few specimens failed at the end caps, likely due to fabrication defects or misalignment that created local stress concentrations and leakage paths. SEM analysis of the MRE membrane 11b revealed microcracks, but these cannot be conclusively identified as the failure origins.

**Table 4:** Performance comparison of the proposed MRE phase-change actuator with similar soft actuators. All performance metrics for the current work correspond to the blocked force configuration (zero strain).

Actuator	Blocked Force(N)	Mass(g)	Untethered	Response Time (s)	Peak Power (W)
Mirvakili et al.^13^	8 N	NA	Yes	110 s	< 100 W
Ai et al.^24^	6.3 N	2.4 g	Yes	200–400 s	NA
This work (MRE)	70 N	23 g	Yes	46 s	150 W

To demonstrate the working of the actuator, a 3 kg load is suspended at one end of the MRE actuator inside a 127 kHz induction coil operated by a 12 V power supply (See supplementary video).

While our proof-of-concept MRE actuator design demonstrates higher force output and electromagnetic tunability with alternating magnetic fields, there remains scope for improvement. While the actuator withstands repeated actuation cycles without acute structural fatigue, the working fluid exhibits gradual permeation over prolonged static periods over days; therefore, improved environmental sealing may help extend its long-term operating shelf life. The response time of 10–20 s is comparatively fast for phase-change actuators, but it remains slower than conventional pneumatic systems, suggesting that further optimization of the MRE composition and phase-change mechanism may be beneficial. Additionally, the system requires high current and operates at an efficiency below 1%, which is typical for thermal actuators^13^. The primary energy losses stem from resistive heating within the induction coil and significant magnetic flux leakage. Specifically, the sparse suspension of ferromagnetic particles allows much of the generated magnetic field to pass through non-conductive domains without performing useful heating work. Future designs can improve efficiency by integrating magnetic flux concentrators to better direct the magnetic field into the fluid chamber.

## Conclusion

This work presented the design and multiphysics analysis of a novel magnetorheological elastomer (MRE)–based untethered artificial muscle actuator. The key contributions of this work can be summarized as follows:

- The MRE-based actuator operating under a 150 kHz excitation field exhibited a faster response and achieved a maximum axial load of 70 N with an actuator mass of 23 g, indicating a high specific force output.
- Finite element modeling quantified the volumetric loss density due to the induction coil and showed its dependence on particle size and excitation frequency, guiding the actuator design.
- Induction coil geometry and operating frequency were found to influence both heating and cooling rates. Increasing frequency from 127 kHz to 150 kHz reduced the actuation time by approximately 25%, demonstrating that actuation dynamics can be tuned through electromagnetic drive parameters.
- The MRE actuator achieved 48% higher blocked load capacity compared to hyperelastic baseline (70 N vs 46 N) and exhibited faster force development across all actuation cycles, indicating improved actuation dynamics in the MRE-based actuator.
- A reduced volume of Novec 7100 resulted in significantly faster actuation, suggesting that actuator dynamics can be adjusted by varying the amount of working fluid.
- By minimizing the peripheral components and removing the need for pumps, valves, and pressure vessels, the actuator enables lightweight, untethered operation, and offers potential for wearable soft robotic applications.

In summary, the proposed induction-driven MRE actuator combines a high force-to-weight ratio and with frequency-controlled actuation dynamics, making it a possible candidate for integration into exosuits and other compact soft robotic systems.

## Data Availability

The datasets generated and/or analysed during the current study are available in the repository: https://github.com/Devansh-IITD25/Phase-Change-MRE. The repository contains the processed experimental data and supplementary video demonstration used in this study. The COMSOL model inputs are provided in the manuscript.
